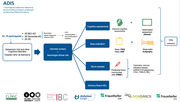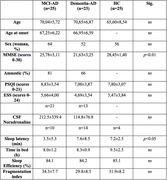# The ADIS study: Early Diagnosis of Alzheimer's Disease by Immune Profiling of Cytotoxic Lymphocytes and Recording of Sleep Disturbances

**DOI:** 10.1002/alz.086465

**Published:** 2025-01-09

**Authors:** Andrea Val‐Guardiola, Neus Falgàs Martínez, Sophia Krix, Vanessa Lage‐Rupprecht, Uri Nevo, Stefan Kirsch, Eti Yoles, Adrià Tort‐Merino, Mircea Balasa, Anna Antonell, Albert Lladó, Kuti Baruch, Soraya Moradi, Christophe Bintener, Nagy István, Holger Fröhlich, Raquel Sanchez‐Valle

**Affiliations:** ^1^ Alzheimer's disease and other cognitive disorders unit, Hospital Clínic, IDIBAPS, Barcelona Spain; ^2^ Fraunhofer Institute for Algorithms and Scientific Computing, Sankt Augustin, NRW Germany; ^3^ Fraunhofer Institute for Algorithms and Scientific Computing, Sankt Augustin Germany; ^4^ Tel Aviv University, Tel Aviv Israel; ^5^ Fraunhofer Institute for Toxicology and Experimental Medicine, Hannover Germany; ^6^ ImmunoBrain Checkpoint Ltd., Ness Ziona Israel; ^7^ Alzheimer’s disease and other cognitive disorders Unit. Hospital Clínic de Barcelona; FRCB‐IDIBAPS; University of Barcelona, Barcelona Spain; ^8^ Alzheimer’s disease and other cognitive disorders Unit. Hospital Clínic de Barcelona. Fundació de Recerca Clínic Barcelona – IDIBAPS. University of Barcelona, Barcelona Spain; ^9^ Hospital Clínic de Barcelona ‐ Fundació de Recerca Clínic Barcelona – IDIBAPS ‐ University of Barcelona, Barcelona, Catalonia Spain; ^10^ Alzheimer Europe, Luxembourg Luxembourg; ^11^ Seqomics, Csongrád Hungary; ^12^ Fraunhofer Institute for Algorithms and Scientific Computing SCAI, Sankt Augustin Germany

## Abstract

**Background:**

Sleep‐wake alterations are common symptoms in Alzheimer’s Disease (AD) associated with faster cognitive decline. Noradrenaline dysfunction and neuroinflammation have been proposed as potential driving mechanisms. The ADIS project aims to study the relationship between sleep‐wake patterns, immune signatures (peripheral blood cytotoxic lymphocytes), and noradrenergic markers across the AD spectrum.

**Method:**

The project aims to recruit a total of 75 participants from the Hospital Clínic de Barcelona, including healthy controls (with normal values of plasma pTau), mild cognitive impairment due to AD (MCI), and AD Dementia (AD) confirmed by CSF biomarkers (Figure 1). All subjects will complete a comprehensive neuropsychological battery and Altoida digital assessment. Sleep‐wake phenotypes will be assessed by sleep questionnaires (Pittsburg Sleep Quality Questionnaire, PSQI, and Epworth Sleepiness Scale, ESS) and 2‐week actigraphy (Motion Wath 8 device). Noradrenaline levels and neuroinflammation markers in CSF will be measured. Immune phenotyping (cytometry, immune activation assays, scRNA/TCR sequencing) of peripheral blood mononuclear cells (PBMC) will be performed. In addition, selected participants will participate on the study's Advisory Board.

**Result:**

To date, we have recruited 25 healthy controls, 25 MCI, and 23 AD participants who performed a total of 67 neuropsychological testings, 47 Altoida assessments, 34 CSF noradrenaline measurements, 65 actigraphy monitoring, and 59 isolation of PBMC. There were no age or gender differences between groups (Table 1). As expected, MMSE was lower in MCI/AD than controls (p<0.01). PSQI and ESS showed no differences, but preliminary analyses of 28 (10 HC, 14 MCI, 4 AD) actigraphy recordings showed that MCI and AD had a greater sleep latency than controls (p<0.05). As expected, preliminary analyses of 34 participants, including 21 MCI and 13 AD, showed similar noradrenaline levels. Further analyses will be performed, including the correlation between immune/noradrenaline markers and clinical data in an increased sample size.

**Conclusion:**

Sleep questionnaires might have a poor performance in identifying sleep alterations in MCI/AD individuals. Digitally assessed sleep‐wake patterns might help to detect sleep alterations. A deeper understanding of the role of biological mechanisms, such as immunologic profiles or noradrenergic dysfunction, driving sleep alteration is warranted.